# *In vivo* overexpression of X-linked inhibitor of apoptosis protein protects against neomycin-induced hair cell loss in the apical turn of the cochlea during the ototoxic-sensitive period

**DOI:** 10.3389/fncel.2014.00248

**Published:** 2014-09-15

**Authors:** Shan Sun, Mingzhi Sun, Yanping Zhang, Cheng Cheng, Muhammad Waqas, Huiqian Yu, Yingzi He, Bo Xu, Lei Wang, Jian Wang, Shankai Yin, Renjie Chai, Huawei Li

**Affiliations:** ^1^Research Center, Affiliated Eye and ENT Hospital of Fudan UniversityShanghai, China; ^2^Department of Otorhinolaryngology, Affiliated Eye and ENT Hospital of Fudan UniversityShanghai, China; ^3^Key Laboratory for Developmental Genes and Human Disease, Ministry of Education, Institute of Life Sciences, Southeast UniversityNanjing, China; ^4^Anesthesiology Department, Xin Hua Hospital Affiliated to Shanghai Jiao Tong University School of MedicineShanghai, China; ^5^Institute of Stem Cell and Regeneration Medicine, Institutions of Biomedical Science, Fudan UniversityShanghai, China; ^6^State Key Laboratory of Genetic Engineering, MOE Key Laboratory of Contemporary Anthropology, School of Life Sciences, Fudan UniversityShanghai, China; ^7^Department of Otolaryngology, The Sixth Hospital Affiliated to Shanghai Jiao Tong UniversityShanghai, China; ^8^State Key Laboratory of Medical Neurobiology, Fudan UniversityShanghai, China

**Keywords:** caspase, apoptosis, IAP, XIAP, aminoglycosides, hair cell loss, hearing loss

## Abstract

Aminoglycoside-induced cochlear ototoxicity causes hair cell (HC) loss and results in hearing impairment in patients. Previous studies have developed the concept of an ototoxicity-sensitive period during which the cochleae of young mice are more vulnerable to auditory trauma than adults. Here, we compared neomycin-induced ototoxicity at the following four developmental ages in mice: postnatal day (P)1–P7, P8–P14, P15–P21, and P60–P66. We found that when neomycin was administered between P8 and P14, the auditory brainstem response threshold increase was significantly higher at low frequencies and HC loss was significantly greater in the apical turn of the cochlea compared to neomycin administration during the other age ranges. Quantitative real-time PCR (qPCR) data revealed that the expression of apoptotic markers, including *Casp3* and *Casp9*, was significantly higher when neomycin was injected from P8 to P14, while the expression of the X-linked inhibitor of apoptosis protein (XIAP) gene was significantly higher when neomycin was injected from P60 to P66. Because XIAP expression was low during the neomycin-sensitive period, we overexpressed XIAP in mice and found that it could protect against neomycin-induced hearing loss at low frequencies and HC loss in the apical turn of the cochlea. Altogether, our findings demonstrate a protective role for XIAP against neomycin-induced hearing loss and HC loss in the apical turn of the cochlea during the ototoxic-sensitive period, and suggest that apoptotic factors mediate the effect of neomycin during the ototoxic-sensitive period.

## Introduction

Aminoglycosides can be ototoxic when administered to adults, children, and infants. It has been reported that the incidence of aminoglycoside-induced cochlear ototoxicity in neonates is greater than that in adults. In addition, the risk of ototoxicity is higher in preterm neonates than in full-term neonates (Henley and Rybak, 1993). The concept of an ototoxicity-sensitive period was first proposed in the 1980s (Chen and Aberdeen, 1981; Chen and Saunders, 1983; Eggermont, 1986) as a time period during which the cochleae of young animals are more vulnerable to auditory traumas (Eggermont, 1986; Henley and Rybak, 1995; Henley et al., 1996). However, the mechanisms involved in the ototoxicity-sensitive period are not well understood.

Several hypotheses have been proposed to describe the hypersensitivity of neonates to ototoxic drugs, and caspase-mediated apoptosis is a common theme (Forge and Li, 2000; Matsui et al., 2002). As one of the primary apoptosis executioner molecules (Ashkenazi and Dixit, 1998; Debatin and Krammer, 2004; Jiang and Wang, 2004; Salvesen and Riedl, 2008), caspase-3 has been widely used to assess the apoptosis of hair cells (HCs) in aminoglycoside-induced ototoxicity (Forge, 1985; Nakagawa et al., 1997; Cunningham et al., 2002; Wei et al., 2005; Tabuchi et al., 2007). Thus, caspase inhibition as a method to prevent cochlear HC death might be a useful treatment strategy for aminoglycoside-induced ototoxicity.

Inhibitor of apoptosis protein (IAP) prevents apoptosis by blocking the classic caspase-mediated apoptotic cascade and the JNK pathway. X-linked inhibitor of apoptosis protein (XIAP) is the most potent IAP and is a broad-range suppressor of apoptosis that functions by directly inhibiting caspases (Deveraux and Reed, 1999; Deveraux et al., 1999). XIAP is broadly expressed in all human tissues except peripheral blood leukocytes, and XIAP overexpression increases the survival of many cell types upon exposure to a variety of apoptotic triggers (Emamaullee et al., 2005; Zhu et al., 2007; Hu et al., 2010; Plesner et al., 2010; Wang et al., 2010, 2011; Unsain et al., 2013).

In this study, we first compared neomycin-induced hearing loss and HC loss at different developmental stages in mice. We then measured the expression levels of apoptosis-related genes in response to neomycin administration in mice at different developmental stages. Finally, we used XIAP overexpression mice to investigate the mechanisms through which XIAP and downstream apoptotic factors affect neomycin-induced ototoxicity during the sensitive period.

## Materials and methods

### Mouse models and treatments

Transgenic mice overexpressing XIAP (Wang et al., 2010, 2011) were kind gifts from Dr. Robert G. Korneluk at the Children's Hospital of Eastern Ontario Research Institute (Ottawa, Ontario, Canada). Experiments were performed in C57BL/6J wild-type (WT) mice and in XIAP overexpression mice (Experimental Animal Center, Shanghai Medical College of Fudan University, China). Postnatal day (P)0 was defined as the day of birth. Mice received a daily subcutaneous injection of neomycin (200 mg/kg) or sterile saline for 7 days. All animal procedures were performed according to protocols approved by the Animal Care and Use Committee of Fudan University and were consistent with the National Institutes of Health Guide for the Care and Use of Laboratory Animals. All efforts were made to minimize the number of animals used and prevent their suffering.

### Auditory brainstem response (ABR) test

The hearing thresholds of the mice were examined with the ABR test. In this test, changes in the electrical activity of the brain in response to sound were recorded via electrodes that were placed on the scalp of the mice. Animals were anesthetized with ketamine (100 mg/kg) and xylazine (25 mg/kg) and placed on a thermostatic heating pad in a sound-attenuating chamber to maintain their body temperatures at 38°C. Frequency-specific auditory responses were measured using the Tucker-Davis Technology system (TDT System III, Alachua, FL, USA) as previously described (Wang et al., 2010). All ABR tests were performed on mice older than P21.

### Tissue preparation

After sacrificing the mice, the right otic capsule was immediately isolated, rapidly frozen in liquid nitrogen, and stored at −70°C until further processing. To obtain the total RNA and protein extract, 10 cochleae were pooled in ice-cold buffer and processed with the Qiagen AllPrep® DNA/RNA/Protein Mini kit following the manufacturer's instructions. The RNA concentration was measured with a Bio-Rad spectrophotometer. Complementary (c)DNA was synthesized from 1 μg total RNA by reverse transcription using random primers (Promega) and Superscript III reverse transcriptase (Life Technologies, Foster City, CA, USA) following the manufacturer's protocols. Quantitative real-time PCR (qPCR) was performed using SYBR Green PCR Master Mix (Life Technologies) on a Bio-Rad IQ5 Detection System (Bio-Rad, USA). Primer sequences are listed in Table 1. The left temporal bones were collected and stored overnight in 4% paraformaldehyde before decalcification in EDTA [4% in phosphate-buffered saline (PBS), pH = 6.4] for a total of 72 h. The otic capsule was then removed, and the cochlea was carefully isolated from the surrounding bony tissue. The organ of Corti was separated and immediately processed for immunofluorescent staining.

**Table 1:** **PCR primer sequences used in the experiments**.

**Gene**	**Forward sequence**	**Reverse sequence**
*Xiap* NM 009688	5′-TTCCCAAATTCAACAAACTCTCCA-3′	5′-ACTTCACTTTATCGCCTTCACCTA-3′
*Diablo* NM023232	5′-CTGGGGGCCCTGGGGTCCTA-3′	5′-GAAAACTGGTCCCTGGTGGTCA-3′
*Xaf1* NM001037713	5′-CTGCCTGCGCTTCATAGTCCTT-3′	5′-TGCACGGCCAGCTCACAGAAC-3′
*Htra2* NM 019752	5′-AGTAGGGCGGGCGAGGAGAGT-3′	5′-AGCAGAGCCCGGAGGTCAGG-3′
*Casp3* NM009810	5′-CGGGGTACGGAGCTGGACTGT-3′	5′-ATGCTGCAAAGGGACTGGATGAAC-3′
*Casp 9* NM 015733	5′-AGGCCCGTGGACATTGGTTCT-3′	5′-AGTTGGAGCCCGTGCGTGTG-3′
β-actin NM_007393.3	5′-TGACGGCCAGGTCATCACTA-3′	5′-ACGGATGTCAACGTCACACTTC-3′

### Immunofluorescence

After fixation, cochlear samples were blocked with 10% normal donkey serum in 10 mM PBS (pH 7.4) with 0.3% Triton-X100 for 1 h at room temperature (RT) and then incubated with primary antibody overnight at 4°C. The next day the tissues were incubated for 2 h at 4°C with 488- or 594-conjugated donkey secondary antibody (1:500 dilution, Invitrogen) and 4,6-diamidino-2-phenylindole (DAPI, 1:800 dilution, Sigma-Aldrich). Omission of primary antibody served as the negative control. The following primary antibodies were used: anti-myosin VIIA (myo7a) (1:500 dilution, cat. #25-6790 Proteus BioSciences), and anti-cleaved caspase-3 (1:1000 dilution, cat. #9579S, Cell Signaling Technology).

Cochleae were dissected into apical, middle, and basal turns and images were taken using a Leica SP5 confocal fluorescence microscope (Leica, Germany).

### Cell counts

For HC quantification in the neomycin-treated samples, we imaged the entire cochlea using a 63× objective and counted the myo7a+ HCs that remained. The same procedure was used to quantify caspase-3+ and caspase-3+/myo7a+ cells. For all experiments, only one cochlea from each mouse was used for immunofluorescence and quantification. Thus, *n* represents the number of mice examined.

### Statistical analyses

Data were expressed as mean ± SD. ABR thresholds were analyzed by a two-way ANOVA followed by a Newman–Keuls *post-hoc* test. Immunofluorescence analysis was performed with a two-tailed, unpaired Student's *t*-test when comparing two groups or with a one-way ANOVA followed by a Dunnett's multiple comparisons test when comparing more than two groups. *p* < 0.05 was considered statistically significant.

## Results

### Neomycin treatment at different stages induced different levels of hearing loss

**Figure 1:**
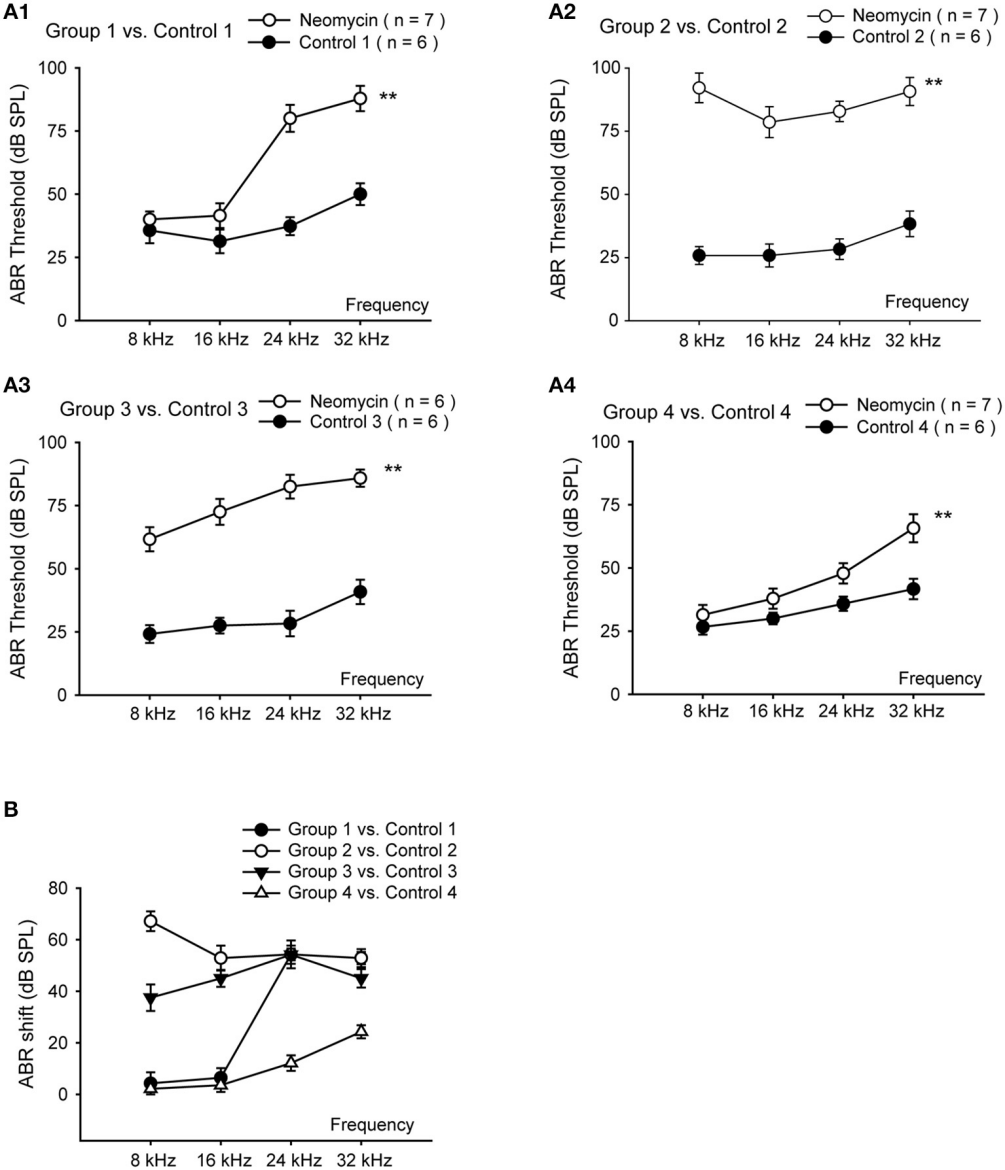
**Hearing loss in neomycin-treated WT mice. (A)** ABR measurements of WT mice 2 weeks after the last injection of neomycin or saline (control). Neomycin or saline was injected once daily between P1 and P7 (Group 1) **(A1)**, P8 and P14 (Group 2) **(A2)**, P15 and P21 (Group 3) **(A3)**, or P60 and P66 (Group 4) **(A4)**. **(B)** ABR threshold shifts of neomycin-treated samples compared to control. ^**^p < 0.01 vs. control, *n* = 6–7.

Aminoglycoside-induced cochlear toxicity in neonatal mice is greater than that in adults (Chen and Aberdeen, 1981; Chen and Saunders, 1983). Here we divided the WT mice into four groups for daily subcutaneous injections of neomycin (200 mg/kg) or saline. Group 1 was injected from P1 to P7, Group 2 was injected from P8 to P14, Group 3 was injected from P15 to P21, and Group 4 was injected from P60 to P66. We measured hearing function using pure tone ABR thresholds in the neomycin-treated and control mice 2 weeks after the last injection. At low frequencies, such as 8 kHz and 16 kHz, ABR thresholds were significantly increased in neomycin-treated mice compared to controls for Groups 2 and 3 (*p* < 0.01 for both frequencies and both groups; *n* = 6–7). However there was no significant difference between the neomycin-treated mice and controls for Groups 1 and 4 (*p* = 0.194 and *p* = 0.056 for 8 and 16 kHz, respectively, in Group 1 and *p* = 0.218 and *p* = 0.145, respectively, in Group 4; *n* = 6–7 mice). At high frequencies, such as 24 and 32 kHz, ABR thresholds were significantly increased in all four groups of neomycin-treated mice compared to controls (*p* < 0.01, *n* = 6–7) (Figures 1A1–A4). Among all four groups, the increase in hearing thresholds in the neomycin-treated mice compared to controls was greatest in Group 2. At 8 kHz, the ABR thresholds in Group 2 increased significantly more than any other group (*p* < 0.01, *n* = 6–7); at 16 kHz, Groups 2 and 3 increased more than the other two groups (*p* < 0.01 for both groups, *n* = 6–7); and at 24 kHz and 32 kHz, Groups 1–3 increased more than Group 4 (*p* < 0.01, *n* = 6–7)(Figure 1B).

### Neomycin treatment at different stages induced different levels of HC loss

**Figure 2:**
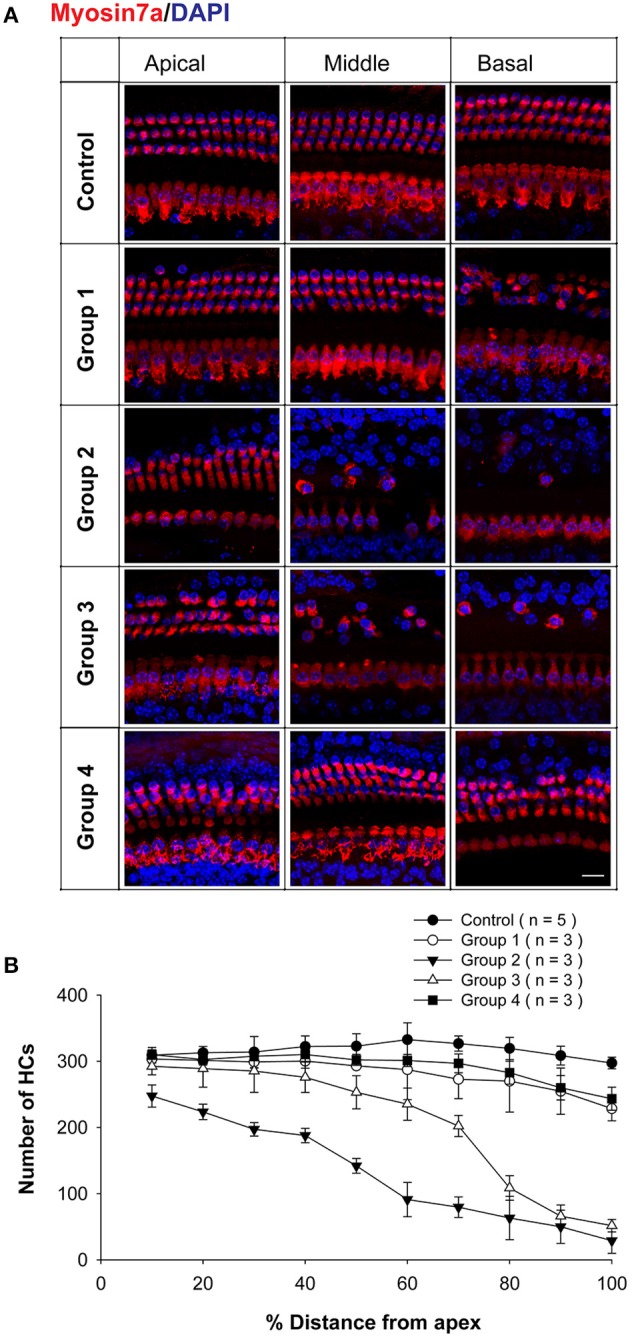
**HC loss in neomycin-treated WT mice. (A)** Representative confocal images of myo7a immunofluorescence in cochlear whole mounts of WT mice treated with neomycin or saline (control) once daily between P1 and P7 (Group 1), P8 and P14 (Group 2), P15 and P21 (Group 3), or P60 and P66 (Group 4) and sacrificed 2 weeks after the last injection of neomycin or saline. **(B)** Myo7a+ HC quantification in WT mice treated with neomycin or saline (control) and sacrificed 2 weeks after the last injection of neomycin or saline. Scale Bar in **A** = 10 μm.

To further investigate the neomycin-induced ototoxicity in the four groups, HC loss in all three turns of the cochlea was evaluated in 3–5 cochleae from each group. Samples were compared 2 weeks after the last injection of neomycin or saline (Figure 2). We found that the number of remaining HCs in Group 2 was significantly lower in all three turns compared to all other groups (*p* < 0.01, *n* = 3–5). In the apical turn, there was no significant difference in the number of remaining HCs among Groups 1, 4, and saline controls (*n* = 3–5). In the middle and basal turns, the number of remaining HCs in Groups 2 and 3 was significantly lower than Groups 1, 4, and saline controls (*p* < 0.01 for both groups, *n* = 3–5). In the basal turn, the number of remaining HCs in Groups 1 and 4 were significantly lower than the saline control group (*p* < 0.01 for both groups, *n* = 3–5) (Figure 2B).

These results showed that Groups 2 and 3 had significant HC loss in the apical turn—while the other groups had no significant HC loss—and that Group 2 in particular had significantly more HC loss in the middle and basal turns than all other groups. This also suggests that HC loss in the apical turn of Group 2 was caused by the hypersensitivity to neomycin-induced ototoxicity during the sensitive period.

### Gene expression levels of apoptosis factors were different when neomycin treatment occurred at different ages

**Figure 3:**
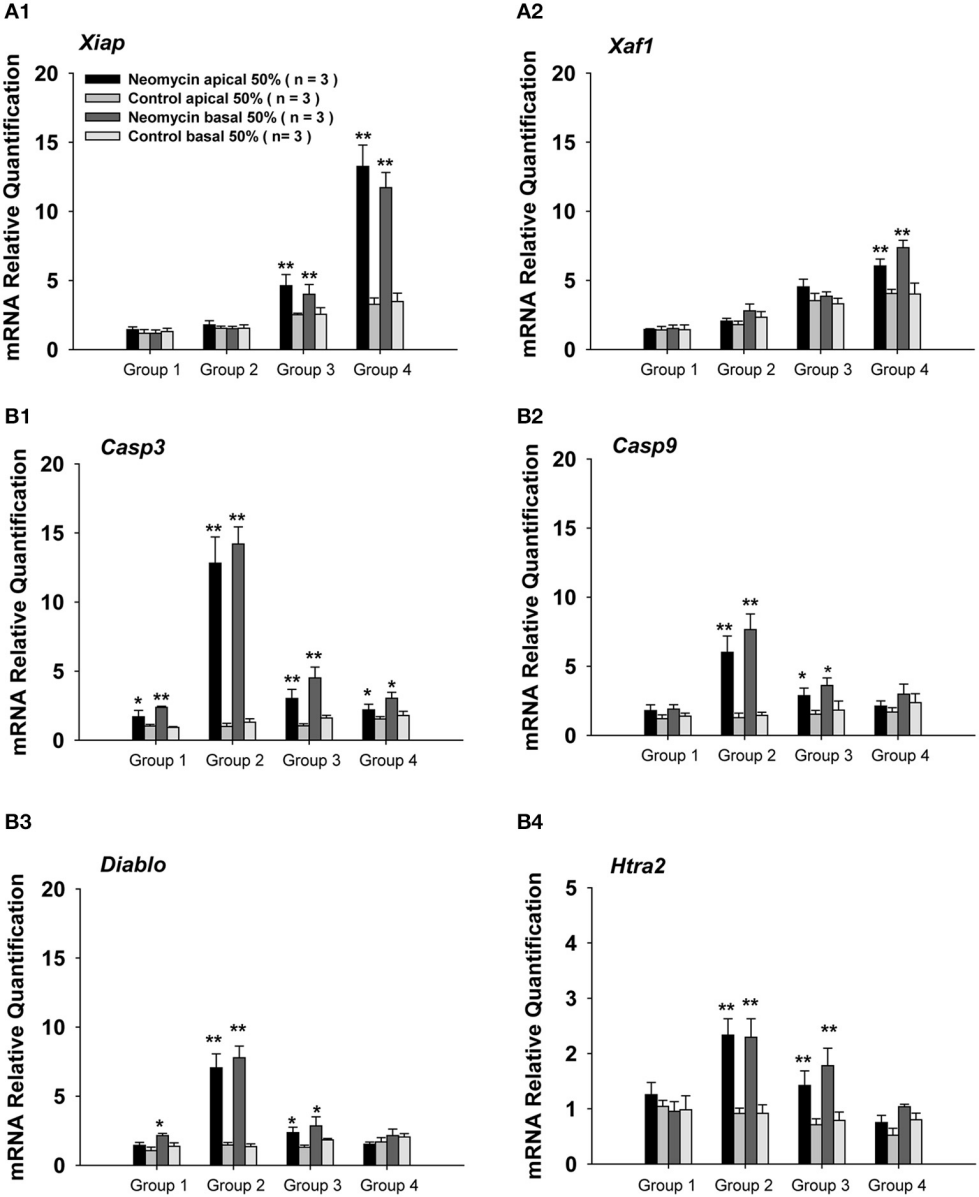
**Apoptosis-related gene expression in neomycin-treated WT mice. (A,B)** mRNA expression of the anti-apoptotic genes *Xiap* **(A1)** and *Xaf1* **(A2)** and the pro-apoptotic genes *Casp3* **(B1)**, *Casp9* **(B2)**, *Diablo* **(B3)**, and *Htra2* **(B4)** in the apical 50% and basal 50% of the cochleae in WT mice sacrificed 3 days after the last injection of neomycin or saline (control). Neomycin or saline was injected between P1 and P7 (Group 1), P8 and P14 (Group 2), P15 and P21 (Group 3), or P60 and P66 (Group 4). ^*^p < 0.05, ^**^p < 0.01 vs. control, *n* = 3.

**Figure 4:**
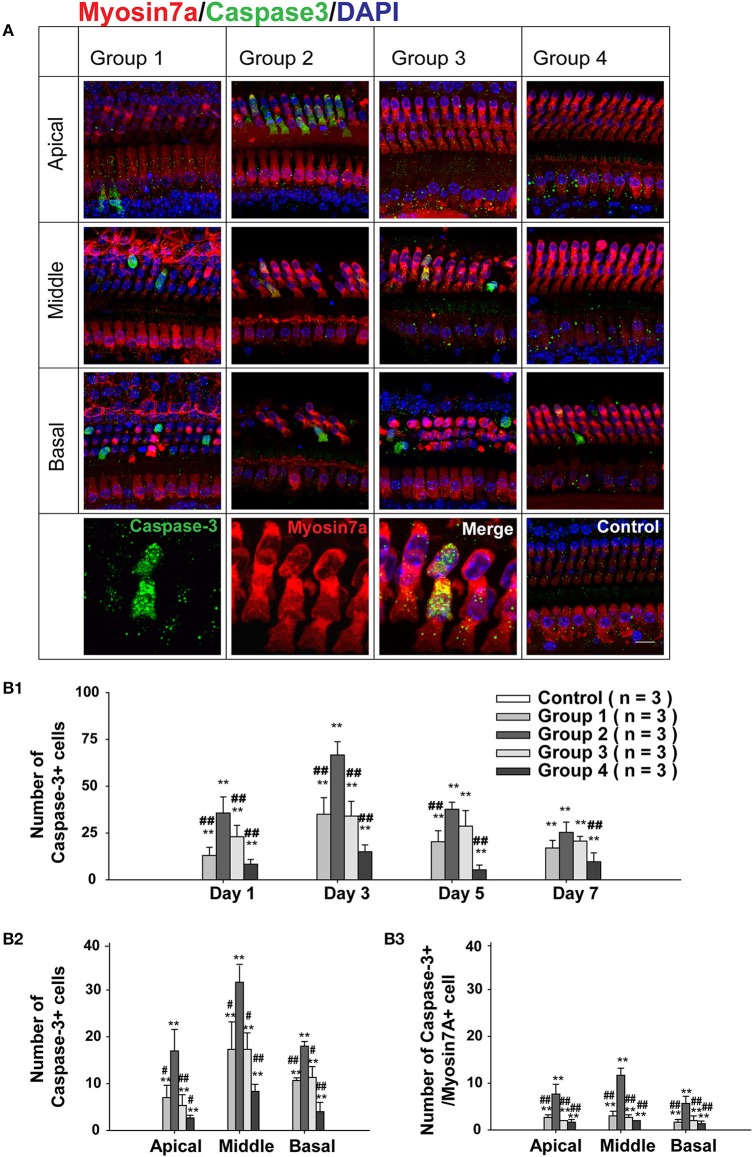
**Caspase-3 expression after neomycin treatment. (A)** Representative confocal images of all three turns of the cochlea stained with myo7a and cleaved caspase-3 in WT mice sacrificed 3 days after the last injection of neomycin or saline (control). Neomycin or saline was injected between P1 and P7 (Group 1), P8 and P14 (Group 2), P15 and P21 (Group 3), or P60 and P66 (Group 4). **(B1)** The number of caspase-3+ cells in the whole cochlea of WT mice treated with neomycin or saline (control) between P1 and P7 (Group 1), P8 and P14 (Group 2), P15 and P21 (Group 3), or P60 and P66 (Group 4) and sacrificed 1, 3, 5, or 7 days after neomycin or saline treatment. **(B2,B3)** The number of caspase-3+ cells **(B2)** and caspase-3+/myo7a+ cells **(B3)** in the three cochlear turns of WT mice treated with neomycin or saline (control) between P1 and P7 (Group 1), P8 and P14 (Group 2), P15 and P21 (Group 3), or P60 and P66 (Group 4) and sacrificed 3 days after the last injection of neomycin or saline treatment. ^**^p < 0.01 vs. control and ^#^*p* < 0.05, ^##^*p* < 0.01, vs. Group 2, *n* = 3. Scale bar in **A** = 10 μm.

To further understand the mechanism resulting in the sensitive period for neomycin-induced ototoxicity, we investigated the gene expression of apoptosis-related genes by qPCR. In this set of experiments, we divided the WT mice into four groups as above and the mice were injected with neomycin or saline for 7 days and sacrificed 3 days after the last injection. The day of sacrifice was determined by cleaved-caspase-3 expression in the cochlea as shown by the immunofluorescence in Figure S1A. The number of caspase-3+ cells reached its peak at 3 days after the last injection (**Figure 4B1**). Because the ABR and HC quantification results showed differences between apical and basal turns, we separated the cochlea into two parts, the apical 50% and the basal 50%. We measured the gene expression of the following anti-apoptotic genes: *Xiap* (X-linked inhibitor of apoptosis), which belongs to a family of apoptotic suppressor proteins, and *Xaf1* (XIAP associated factor 1), which binds to and counteracts the inhibitory effect of a member of the IAP (inhibitor of apoptosis) protein family. We also measured the expression levels of the following pro-apoptotic genes: *Casp3* and *Casp9*, which are members of the caspase family and play central roles in the execution-phase of apoptosis; *Diablo*, which encodes an IAP-binding protein; and *Htra2*, which encodes a serine protease that induces apoptosis by binding to the apoptosis inhibitory protein XIAP. All of the gene expression data were normalized to the gene expression of P3 cochleae treated with saline. When compared to saline controls, we found that the expression levels of the anti-apoptotic genes *Xiap* and *Xaf1* were significantly increased in neomycin-treated cochleae from Groups 3 and 4 (*p* < 0.01 for both groups, *n* = 3) and that the expression levels in the apical 50% of the cochlea were slightly higher than in the basal 50%. There was no significant increase of *Xiap* or *Xaf1* in neomycin-treated cochleae in Groups 1 and 2 compared to their saline controls (Figure 3A), and this indicated that *Xiap* might protect the cochlea from neomycin-induced ototoxicity. Compared to saline controls, the expression levels of the apoptotic genes *Casp3*, *Casp9*, *Diablo*, and *Htra2* were significantly increased in neomycin-treated cochleae in Groups 2 and 3 (*p* < 0.01 for both groups, *n* = 3). The expression of *Casp3* was also significantly increased in Groups 1 and 4 (*p* < 0.01 for both groups, *n* = 3), but the expression levels of the other three apoptotic genes were only slightly increased in Groups 1 and 4. In addition, the expression of apoptotic genes in the apical 50% of the cochlea was slightly lower than the basal 50% (Figure 3B). When compared among the four groups, we found that the expression levels of the anti-apoptotic genes *Xiap* and *Xaf1* increased with age in saline controls. After neomycin exposure, *Xiap* and *Xaf1* expression levels in Groups 1 and 2 were similar to controls. In Group 4, however, the expression levels of *Xiap* and *Xaf1* were significantly higher than all other groups (Figure 3A). Because XIAP has protective effects against apoptosis, one underlying cause of the sensitive period for neomycin-induced ototoxicity might be low XIAP levels. We also found that the expression levels of pro-apoptotic genes *Casp3*, *Casp9*, *Diablo*, and *Htra2* were significantly higher in Group 2—in which neomycin was injected during the ototoxicity-sensitive period of P8 to P14—than in all other groups (Figure 3B), and this suggests that apoptotic factors are involved in the sensitive period for neomycin-induced ototoxicity.

### Caspase-mediated apoptosis was involved in the sensitive period of neomycin-induced ototoxicity

To confirm the involvement of caspase-mediated apoptosis in the postnatal sensitive period for neomycin-induced ototoxicity *in vivo*, we also divided the WT mice into four groups as described above, injected them with neomycin or saline for 7 days, sacrificed the mice at 1, 3, 5, or 7 days after the last injection, and stained the cochleae with cleaved-caspase-3 and myo7a antibodies (Figure S1A). We found that the number of HCs kept decreasing from day 1 to 7 in all neomycin-treated groups. At each time point, there was a significant difference between Group 2 and the three other neomycin-treated groups (*p* < 0.01, Figure S1). Moreover, the number of caspase-3+ cells was greatest at 3 days following the neomycin injection (Figures 4B1, S1A), and this was the time-point chosen for further analysis. At 3 days after neomycin or saline treatment, no caspase-3+ cells were observed in any of the three turns of the control cochleae treated with saline, and this was consistent with a previous report (Kaiser et al., 2008). Among the four groups with neomycin treatment, Group 2 had significantly more caspase-3+ and caspase-3+/myo7a+ cells in the apical turn of cochlea (17.00 ± 4.58 cells and 7.67 ± 2.10 cells per cochlear apical turn, respectively, *p* < 0.01, *n* = 3). The other three groups had very few caspase-3+ cells and almost no caspase-3+/myo7a+ cells in the apical turn (Figures 4A,B2,B3). This result suggested that caspase-mediated apoptosis is involved in the neomycin-induced ototoxicity of the apical turn during the sensitive period. In middle and basal turns, all four groups had significantly more caspase-3+ and caspase-3+/myo7a+ cells than the control groups (*p* < 0.01, *n* = 3), but Group 2 had significantly more caspase-3+ cells (31.67 ± 3.51 cells and 18.33 ± 1.38 cells per cochlear middle and basal turn, respectively) compared to Groups 1, 3, and 4 (17.33 ± 5.86 cells and 10.67 ± 0.58 cells, 17.33 ± 3.52 cells and 11.33 ± 2.31 cells, 8.33 ± 1.52 cells and 4.00 ± 2.13 cells per cochlear middle and basal turn per group, respectively, *p* < 0.01, *n* = 3) and Group 2 had more caspase-3+/myo7a+ cells (11.67 ± 1.53 cells and 5.67 ± 1.53 cells per cochlear middle and basal turn, respectively) compared to Groups 1, 3, and 4 (3.00 ± 1.12 cells and 1.67 ± 0.58 cells, 2.67 ± 0.58 cells and 2.00 ± 1.21 cells, 2.00 ± 0.67 cells and 1.33 ± 0.58 cells per cochlear middle and basal turns per group, respectively, *p* < 0.01, *n* = 3) (Figures 4A,B2,B3). This result suggested that caspase-mediated apoptosis is also involved in the postnatal sensitive period of neomycin-induced ototoxicity in middle and basal turns.

### XIAP overexpression attenuated neomycin-induced HC loss in the apical turn during the ototoxicity-sensitive period

**Figure 5:**
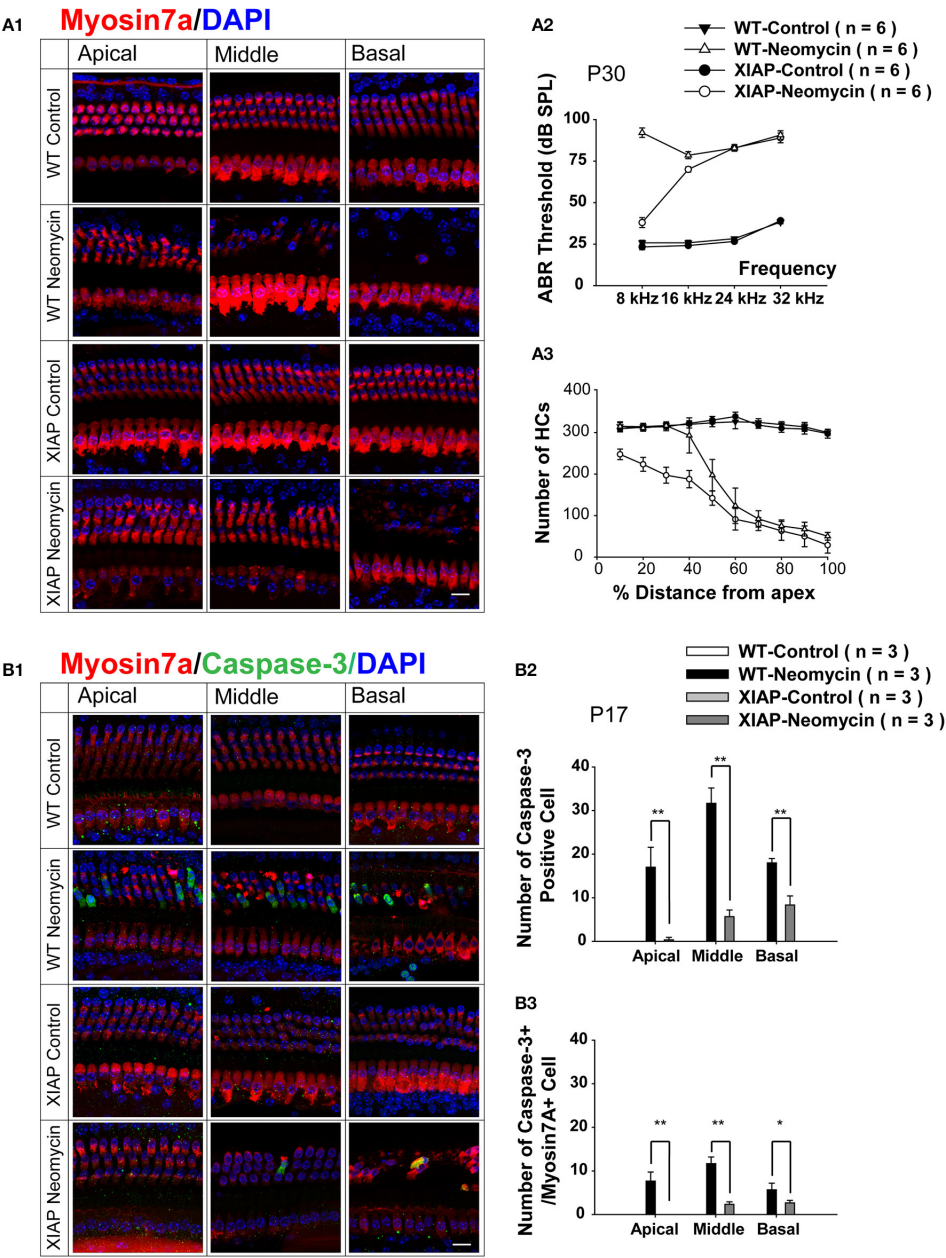
**Hearing loss and HC loss in neomycin-treated XIAP overexpression mice. (A1)** Representative confocal images of myo7a immunofluorescence in cochlear whole mounts of XIAP overexpression and WT mice treated with neomycin or saline (control) between P8 and P14 (Group 2) and sacrificed at P30. ABR measurement **(A2)** and myo7a+ HC quantification **(A3)** of P30 XIAP overexpression and WT mice treated with neomycin or saline (control) between P8 and P14. **(B1)** Representative confocal images of myo7a and cleaved-caspase-3 immunofluorescence in cochlear whole mounts of XIAP overexpression and WT mice treated with neomycin or saline (control) between P8 and P14 (Group 2) and sacrificed at P17. **(B2,B3)** The quantification of caspase-3+ cells (B2) and caspase-3+/myo7a+ cells **(B3)** in the three cochlear turns of XIAP overexpression and WT mice treated with neomycin or saline (control) between P8 and P14 (Group 2) and sacrificed at P17. ^*^p < 0.05, ^**^p < 0.01 vs. WT neomycin, *n* = 3. Scale bar in **A** = 10 μm.

To further investigate whether apoptotic factors are involved in the sensitive period to neomycin-induced ototoxicity in the apical turn, we took advantage of genetic methods to inhibit the apoptotic factors in neomycin-treated mice. Here we used transgenic XIAP overexpression mice, in which the coding sequence of XIAP is under the control of a ubiquitin promoter (ubXIAP) (Wang et al., 2010). In this experiment, we injected WT or ubXIAP overexpression mice with neomycin or saline during the ototoxicity-sensitive period (Group 2, P8–P14). ABR thresholds were measured from six mice in each group at P30, and HC loss in all three cochlear turns was evaluated from 4–5 cochleae in each group at P30 (Figure 5A). In the neomycin-treated groups, ABR thresholds were significantly decreased at 8 kHz in the XIAP overexpression mice compared to WT mice (*p* < 0.01, *n* = 6). There was a slight decrease at 16 kHz (*p* < 0.05, *n* = 6), but no significant difference was detected at 24 and 32 kHz. There was no ABR threshold increase in the XIAP overexpression mice without neomycin injection (Figure 5A2).

Moreover, in the neomycin-treated groups, there was almost no HC loss in the apical turn of the cochleae from XIAP overexpression mice, and this demonstrated that XIAP overexpression almost completely prevented the neomycin-induced HC loss in the apical turn during the ototoxicity-sensitive period. HC loss was slightly attenuated in middle and basal turns in XIAP overexpression mice. There was no HC loss in XIAP overexpression mice without neomycin treatment (Figures 5A1,A3).

We further examined the cleaved-caspase-3 expression in neomycin-treated XIAP overexpressing mice. In this experiment, we injected WT or ubXIAP mice with neomycin or saline from P8 to P14, sacrificed the mice at 3 days after the last injection, and stained the cochleae with cleaved-caspase-3 and myo7a antibodies (Figure 5B1). We found that when treated with neomycin, XIAP overexpression mice had only a very small number of caspase-3+ cells and no caspase-3+/myo7a+ HCs in the apical turn. They also had significantly fewer caspase-3+ cells and caspase-3+/myo7a+ HCs in the middle and basal turns compared to the WT mice (Figures 5B2,B3, *p* < 0.05, *n* = 3). Together, these results suggest that when neomycin was injected during the ototoxicity-sensitive period, HC loss in the apical turn was primarily caused by the hypersensitivity to neomycin-induced ototoxicity (Figure 2). XIAP overexpression significantly reduced hearing loss and HC loss in the apical turn, but only partially prevented the HC loss in the middle and basal turns (Figure 5). We thus conclude that apoptotic factors are involved in the sensitive period of neomycin-induced ototoxicity in the apical turn of the cochlea.

## Discussion

Aminoglycosides are effective against aerobic gram-negative infections and are widely used to treat tuberculosis in people in developing countries and to treat life-threatening bacterial infections in premature infants across the world. All aminoglycosides have the potential for nephrotoxicity, ototoxicity, neuromuscular blockade, allergic reactions, and hematotoxicity, and these adverse effects limit the clinical use of aminoglycoside antibiotics. Aminoglycoside ototoxicity is typically associated with bilateral sensorineural hearing loss and tinnitus (Nadol, 1993), yet there have been only a few *in vivo* investigations of aminoglycoside ototoxicity in mammalian development.

Previous reports indicated that aminoglycoside-induced cochlear HC loss is more severe in neonatal mice than in adults, and this led to the development of the concept of an ototoxicity-sensitive period (Chen and Aberdeen, 1981; Chen and Saunders, 1983; Eggermont, 1986; Henley and Rybak, 1993, 1995; Henley et al., 1996). However the mechanism underlying this sensitive period has not been elucidated. In our experiments, we injected neomycin during the four different ages of P1–P7, P8–P14, P15–P21, and P60–P66 and found that all groups of C57BL/6J WT mice were sensitive to neomycin-induced hearing loss at high frequencies. However, at low frequencies only the P8–P14 group was sensitive to neomycin-induced hearing loss and only that group had significant HC loss in the apical turn. Taken together, these results suggest that HC loss in the apical turn is primarily caused by hypersensitivity to neomycin-induced ototoxicity during the sensitive period of P8 to P14.

Pharmacokinetics experiments have shown that aminoglycosides have a longer serum half-life and a lower serum clearance rate in neonates than in adults, and this might result in increased risk of permanent ototoxic damage in neonates (Henley and Rybak, 1993; Berard et al., 1994; Scaglione et al., 1995). In addition to metabolic and anatomic factors, animal models of aminoglycoside-induced ototoxicity have also indicated hypersensitivity of neonates to ototoxic drugs compared to adults, but the underlying mechanisms behind this hypersensitivity remain unclear. When aminoglycoside antibiotics are taken up into sensory HCs and transported to endosomes and lysosomes, lysosomal phospholipidosis is followed by lysosomal rupture and this lead to the release of aminoglycosides into the cytoplasm and impairment of mitochondrial respiration (Chen et al., 1996). This in turn can lead to several different biochemical events. (1) Complexes can form with transition metals (iron and copper), and these promote the formation of superoxide radicals such as hydroxyls. These in turn can trigger lipid peroxidation that progressively inhibits metabolic activities (Guo et al., 2001). (2) There is increased calcium influx into cells resulting in calcium overload. (3) Caspase-mediated apoptotic cascades are initiated (Forge, 1985; Li et al., 1995; Nakagawa et al., 1997; Cunningham et al., 2002). (4) JNK-mediated cell apoptosis is initiated (Ylikoski et al., 2002; Sugahara et al., 2006). (5) The permeability of the mitochondria changes, and this leads to the release of apoptosis-inducing factor and endonuclease G, both of which can then move into the nucleus and activate caspase-independent pathways that can lead to apoptosis-like features in cells (Jiang et al., 2006).

In our study, HC damage in the basal turn of the cochlea was much more severe than in the apical turn, and this is correlated with previous reports that outer HCs from the basal turn are more susceptible to damage than outer HCs in the apical turn (Sha et al., 2001; Jensen-Smith et al., 2012). The underlying cause for this phenotype might be the following: (1) the cochlear blood flow moves more quickly in the basal turn and this results in higher concentrations of drugs in the basal turn; (2) the outer HCs of the basal turn are smaller and contain more mitochondria and are thus more susceptible to damage; and (3) the distribution and activity of drug transporters in basal and apical outer HCs is different (Ding and Salvi, 2007).

To investigate the underlying mechanism behind the hypersensitivity to neomycin-induced ototoxicity in the apical turn during the sensitive period, we measured the expression level of apoptosis-related genes when neomycin was injected during different periods. We found that the expression levels of the apoptotic factors *Casp3*, *Casp9*, *Diablo*, and *Htra2* were significantly higher when neomycin was injected during the sensitive period compared to other periods, and this suggested that apoptotic factors are involved in the sensitive period for neomycin-induced ototoxicity.

We then investigated the possible molecular biological mechanism leading to the ototoxicity-sensitive period. XIAP has the most potent anti-apoptotic effects. It contains a single C-terminal RING domain and three tandem baculoviral IAP repeat (BIR) domains. BIR1 and BIR2 have been shown to inhibit caspase-3 and caspase-7 activation (Suzuki et al., 2001), and the caspase-9 inhibitory activity of XIAP has been localized to the BIR3-RING domain (Srinivasula et al., 2001; Shiozaki et al., 2003; Zou et al., 2003). It has been well established that overexpression of XIAP can protect against apoptosis. There is also evidence that AAV-mediated delivery of XIAP protects against cisplatin ototoxicity (Cooper et al., 2006). XIAP overexpression has been demonstrated to prevent neuronal death in models of transient cerebral ischemia and Parkinson's disease (Xu et al., 1999; Ishigaki et al., 2002; Crocker et al., 2003; Trapp et al., 2003). The same ubiquitous XIAP overexpression mice used in this study also exhibit later onset of presbycusis and an insensitivity to noise-induced hearing loss (Wang et al., 2010, 2011).

To further investigate the role of XIAP in the sensitive period for neomycin-induced ototoxicity, we injected neomycin into transgenic XIAP overexpression mice during the sensitive period. ABR data showed that XIAP overexpression protected against hearing loss from neomycin-induced ototoxicity at 8 kHz, but the protective effect was not significant at 16, 24, or 32 kHz. When we quantified the number of remaining HCs, we found that XIAP overexpression almost completely protected against HC loss in the apical turn, but this protective effect was not significant in the middle and basal turns. Thus, there might be other non-apoptotic mechanisms involved in HC death in the middle and basal turns as we discussed previously.

All of these results showed that when neomycin was injected during the ototoxicity-sensitive period HC loss in the apical turn was primarily caused by hypersensitivity to neomycin-induced ototoxicity and that XIAP overexpression almost completely prevented this HC loss in the apical turn. We thus conclude that apoptotic factors are involved in the apical turn during the ototoxicity-sensitive period.

In contrast to the protective effect described above, XIAP overexpression failed to protect against hearing loss or against HC loss in middle and basal turns of the cochlea. Our qPCR data showed that *Casp3* expression was significantly elevated in the entire cochlea when neomycin was injected during the ototoxicity-sensitive period, but overexpression of XIAP—the most potent caspase inhibitor—was only able to partially prevent HC loss in the middle and basal turns. One possible reason for this limited protection is that neomycin-induced ototoxicity in the middle and basal turns is not mediated by the classical caspase-induced apoptosis pathway or the JNK signaling pathway, but rather by caspase-independent apoptosis pathways (Jiang et al., 2006) or by necrosis due to the chronic nature of drug administration (Wang and Li, 2000). It is also possible that ubiquitous overexpression of human XIAP in transgenic mice might not be sufficient to protect against the neomycin-induced HC loss in the middle and basal turns where neomycin concentrations are higher.

In summary, our results show that XIAP overexpression can successfully prevent neomycin-induced hearing loss and HC loss in the apical turn of the cochlea during the sensitive period. These results also provide *in vivo* evidence that apoptotic factors are involved in the sensitive period of neomycin-induced ototoxicity. Overexpression of XIAP, therefore, might serve as a novel therapeutic target for reducing aminoglycoside-induced HC damage and hearing loss.

## Author contributions

Shan Sun, Jian Wang, Shankai Yin, Renjie Chai and Huawei Li developed the concepts or approach; Shan Sun, Mingzhi Sun, Yanping Zhang, Cheng Cheng, Muhammad Waqas, Huiqian Yu, Yingzi He, and Bo Xu performed experiments; Shan Sun, Mingzhi Sun, Cheng Cheng, Muhammad Waqas, Lei Wang performed data analysis; Shan Sun, Mingzhi Sun, Lei Wang , Renjie Chai and Huawei Li wrote the manuscripts, Yanping Zhang, Cheng Cheng, Muhammad Waqas, Huiqian Yu, Yingzi He, Bo Xu, Jian Wang and Shankai Yin edited the manuscript.

### Conflict of interest statement

The authors declare that the research was conducted in the absence of any commercial or financial relationships that could be construed as a potential conflict of interest.

## Supplementary material

Figure S1 **Caspase-3 expression in WT mice after neomycin treatment. (A)** Representative confocal images of myo7a immunofluorescence in cochlear whole mounts of WT mice sacrificed at 1, 3, 5, or 7 days after the last injection of neomycin or saline (control). Neomycin or saline was injected between P1 and P7 (Group 1), P8 and P14 (Group 2), P15 and P21 (Group 3), or P60 and P66 (Group 4). **(B)** Myo7a+ HC quantification in WT mice treated with neomycin or saline (control) between P1 and P7 (Group 1), P8 and P14 (Group 2), P15 and P21 (Group 3), or P60 and P66 (Group 4) and sacrificed at 1, 3, 5, or 7 days after the last injection of neomycin or saline. ^**^*p* < 0.01 vs. control and ^#^*p* < 0.05, ^##^*p* < 0.01 vs. Group 2, *n* = 3. Scale bar in **A** = 10 μm.

## Abbreviations

HC: hair cell
P: postnatal day
WT: wild type
ABR: auditory brainstem response
XIAP: X-linked inhibitor of apoptosis protein
IAP: inhibitor of apoptosis protein
Xaf1: XIAP-associated factor 1
AIF: apoptosis-inducing factor
BIR: baculoviral IAP repeat.
