# Supplementary figures and images for: In vivo overexpression of X-linked inhibitor of apoptosis protein protects against neomycin-induced hair cell loss in the apical turn of the cochlea during the ototoxic-sensitive period
Source: Front Cell Neurosci. 2014 Sep 15;8:248. doi: 10.3389/fncel.2014.00248 (PMC4166379; doi:10.3389/fncel.2014.00248)

A

Myosin7a/Caspase3/DAPI

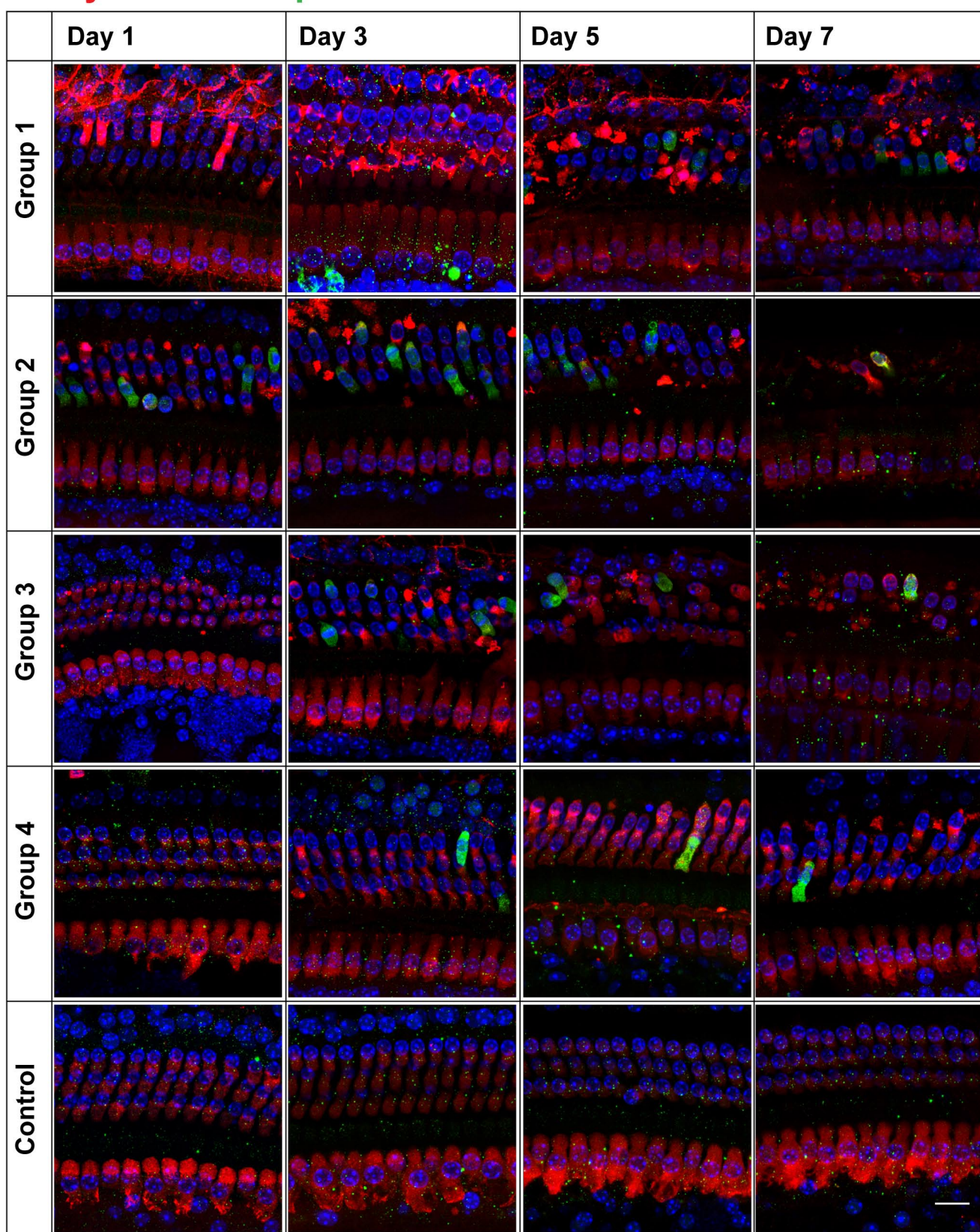

B

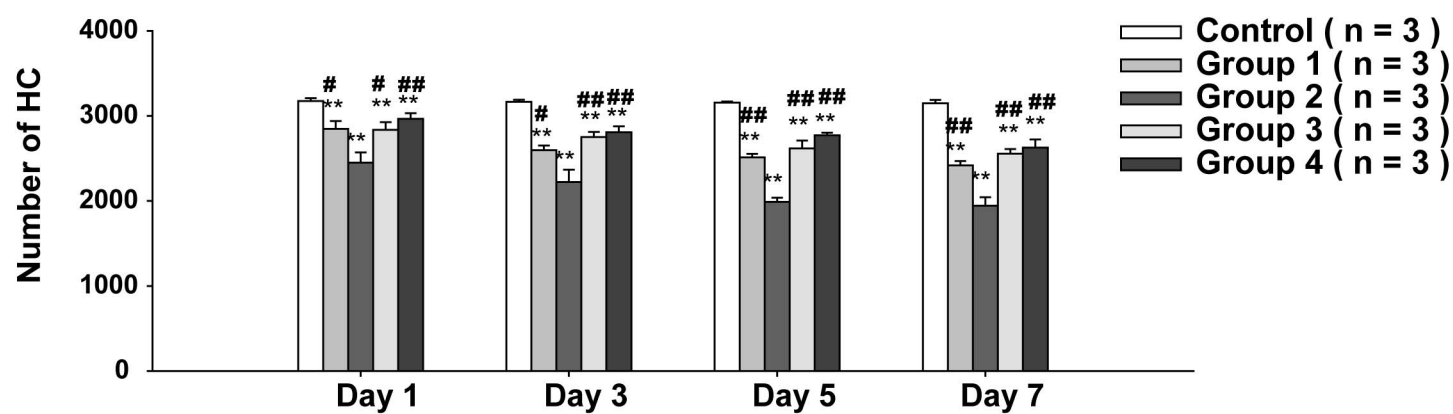

Supplement: Figure S1 — Caspase-3 expression in WT mice after neomycin treatment. (A) Representative confocal images of myo7a immunofluorescence in cochlear whole mounts of WT mice sacrificed at 1, 3, 5, or 7 days after the last injection of neomycin or saline (control). Neomycin or saline was injected between P1 and P7 (Group 1), P8 and P14 (Group 2), P15 and P21 (Group 3), or P60 and P66 (Group 4). (B) Myo7a+ HC quantification in WT mice treated with neomycin or saline (control) between P1 and P7 (Group 1), P8 and P14 (Group 2), P15 and P21 (Group 3), or P60 and P66 (Group 4) and sacrificed at 1, 3, 5, or 7 days after the last injection of neomycin or saline. **p < 0.01 vs. control and #p < 0.05, ##p < 0.01 vs. Group 2, n = 3. Scale bar in A = 10 μm. [file Image1.PDF]
